# Aircraft trajectory prediction and aviation safety in ADS-B failure conditions based on neural network

**DOI:** 10.1038/s41598-023-46914-2

**Published:** 2023-11-11

**Authors:** Zhanji Yang, Xiaolei Kang, Yuanhao Gong, Jiansheng Wang

**Affiliations:** 1 Naval Petty Officer Academy, Bengbu, 233010 China; 2 China Merchants Chongqing Communications Technology Research and Design Institute, Chongqing, 400067 China; 3 Civil Aviation Flight University of China, Guanghan, 618307 China

**Keywords:** Aerospace engineering, Electrical and electronic engineering

## Abstract

With the rapid expansion of transportation demand, the number of global flights has rapidly increased, which also poses challenges to air traffic management (ATM). Considering that the radar system in ATM can no longer meet the requirements of flight safety, a very promising next-generation air traffic control technology—Automatic Dependent Surveillance Broadcast (ADS-B) technology has been introduced. However, in the event of on-board equipment failure and local area signal interference, the ADS-B’s signal will disappear or be interrupted. This sudden situation can pose a danger to aviation safety. To solve this problem, this article proposes a bidirectional long short-term memory (Bi-LSTM) network prediction method combining historical ADS-B data to short-term predict the trajectory of aircraft, which can improve aviation safety in busy airspace. Firstly, the problem of frequent dynamic modeling of different types of aircraft was solved by utilizing historical ADS-B data as the data source. Secondly, the data cleansing method is proposed for ADS-B raw data. Furthermore, considering that the spatial trajectory of the aircraft is a complex time series with continuity and interactivity, a bidirectional LSTM based aircraft trajectory prediction framework is proposed to further improve prediction accuracy. Finally, a trajectory with frequent changes was selected for prediction, and compared with 7 prediction methods. The results showed that the proposed method had high prediction accuracy, thus also improving the aviation safety of the aircraft.

## Introduction

With the continuous advancement of technology, the application scope of aircraft in human society continues to expand. In the civil field, the most typical application is the aircraft used to transport passengers around the world. International Air Transport Association (ITAT) indicated that flight transportation and air passenger demand has remained strong. In the next 20 years, the annual growth rate of global air transport will be about 4.4%, and China's air traffic will increase 3.5 times, which poses a major challenge to the Air Traffic Management (ATM).

Radar systems are currently used by ground monitoring stations to observe aircraft trajectories. However, considering that the radar system in ATM can no longer meet the needs of aviation safety, a very promising next-generation air traffic control (ATC) technology—Automatic Dependent Surveillance Broadcast (ADS-B) technology has been introduced.

The ADS-B does not require manual operation or inquiry like secondary radar, and the aircraft automatically broadcasts its position, altitude, speed, course, identification number and other information to other aircraft or ground stations for the controller and pilots to monitor the status of the aircraft. Considering that the ADS-B data contains latitude, longitude and altitude information, it can be used to observe aircraft trajectories. For example, FlightRadar24 and FlightAware use ADS-B data to provide real-time aircraft tracking, aircraft flight path playback, and global flight path data mapping services.

Currently, most aircraft are equipped with ADS-B equipment in accordance with the Federal Aviation Administration of the United States (FAA) and the Civil Aviation Administration of China (CAAC) standards as the primary means of aviation safety and ATMs. According to data from CAAC, the proportion of integrated application of ADS-B in the air traffic control automation system has increased from 65% at the end of 2019 to 92% by 2022. This not only provides surveillance support for radar free areas in China, but also provides continuous and stable supplementary replacement services multiple times when radar is out of service, promoting a dual improvement in safety level and operational efficiency.

However, in the event of on-board equipment failure and local area signal interference, the ADS-B signal will disappear or be interrupted, which will bring danger to aviation safety. Therefore, in the event of ADS-B failure, it is important to make short-term predictions of the trajectory of the aircraft, which can improve aviation safety in busy airspace.

This article proposes a bidirectional long short-term memory (Bi-LSTM) network prediction method combining historical ADS-B data to short-term predict the trajectory of aircraft, which can improve aviation safety in busy airspace. The main study work is as follows:

1. Since October 10, 2019, the first phase of ADS-B regulatory operation has been carried out in China. China has become the second country in the world to implement ADS-B controlled operations on a large scale in its entire airspace, after Australia. Therefore, the practical application of ADS-B on civil aviation aircraft is a relatively new field. Furthermore, there are still relatively few in-depth studies on its failure scenarios, so this study is novel in addressing this emerging issue.
2. Traditional methods require frequent dynamic modeling of different types of aircraft under different conditions to obtain simulated flight data. This study adopts a data-driven approach and proposes to use historical ADS-B data to predict the aircraft trajectory when ADS-B fails. At the same time, in view of the problem that the original ADS-B data contains some erroneous data, which is not conducive to model training, a data cleaning method for processing ADS-B original data is proposed. It can provide high-quality data sources for the trajectory prediction model of this study and improve the scalability of the method.
3. ADS-B is prone to failure due to intentional or unintentional illegal interference, which in turn leads to interruption of flight monitoring, thereby increasing the risk of accidents. Through accurate trajectory prediction, real-time data can be provided when ADS-B fails, which can immediately strengthen air-ground coordination and linkage, gain prime time for crew members to implement emergency plans, and further ensure flight safety. This study combines neural network technology to propose a Bi-LSTM aircraft trajectory prediction framework, which can predict the aircraft trajectory when ADS-B fails and improve aviation safety in emergencies.

The rest of this paper is organized as follows. Section "Related works" reviews the literature related to aircraft trajectory prediction. Section "Theoretical basis of neural network for predicting aircraft trajectories" presents theoretical basis for predicting aircraft trajectories using neural networks. Section "Aircraft trajectory prediction framework" presents the aircraft trajectory prediction framework proposed in this article. Section "Experiment" presents the experimental results. Concluding remarks are described in Section "Conclusions".

## Related works

At present, study on trajectory prediction of aircraft have been done extensively by related scholars. In order to improve the prediction accuracy of flight trajectory, scholars have developed state estimation model, dynamic model and machine learning model.

### State estimation methods

Kang et al.^1^ used visual sensors to obtain the position and attitude of the air-craft, and then deduced the conventional specific extreme power and an energy maintaining specific power. Finally, these powers were used in the particle dynamics model for trajectory prediction. Yepes et al.^2^ uses a hybrid estimation algorithm to estimate the status and flight mode of an aircraft, and then uses air traffic regulations, aircraft flight plans, and environmental knowledge to infer the pilot's intentions. Finally, aircraft trajectories are predicted based on estimated aircraft status, flight patterns, and inferred pilots' intentions. Liu et al.^3^ proposed an intent based trajectory prediction (IBTP) algorithm. The algorithm first predicts the trajectory based on Interacting Multiple Model (IMM), in which the dynamic model with the heading angle closer to the expected direction is assigned a larger weight. Secondly, pseudo-measurements are generated based on the spatial information in the expected waypoint (for example, location and speed) to smooth the predict-ed trajectory.

Liu et al.^4^ presents a stochastic linear hybrid system to describe the dynamics of an aircraft with varying flight modes. Then, according to the flight plan, the random linear hybrid system is divided into two different discrete state transfer models: Markov transfer model and state-dependent transfer model. Finally, based on the state-dependent transition model, an algorithm for 4-D trajectory prediction of aircraft is presented.

Ayhan et al.^5^ considers the airspace as a 3D mesh network, creates a new form of trajectory with weather information, and then predicts the trajectory with ambient uncertainty using a hidden Markov model (HMM).

Due to the limitations of the air traffic regulations and the aircraft kinematics model, the trajectory of the aircraft is long-distance dependent. Rezaie et al.^6^ proposed a conditional Markov (CM) sequence considering the information of waypoints.

In disaster situations, UAVs and aircrafts will perform rescue missions jointly in limited airspace. However, there is a risk of collision—conflict detection and resolution (CD&R) issues. Wu et al.^7^ established a calculation method of UAV collision prob-ability under the constraints of mission space and the number of UAVs, and solved the flight path conflict problem of high-density UAV clusters. In addition, a state estimation method based on Kalman algorithm is proposed to realize aircraft trajectory planning. Lin et al.^8^ proposed an unmanned aerial vehicle path prediction algorithm to estimate flight trajectories, which can provide early warning before a collision occurs. This study assumes an airspace around a disaster site that requires multiple air support. In this con-text, a hybrid model is built by combining three different prediction conditions, which provides a more accurate CD&R system for manned and unmanned aircraft in cooperative rescue missions.

Lymperopoulos et al.^9^ developed a new particle filter algorithm to improve the ac-curacy of trajectory prediction (TP) by combining the information of multiple aircraft at different positions and times.

Zhang et al.^10^ proposed a modified interacting multiple model (M-IMM) to predict the aircraft trajectory. This model based on the IMM and considering the influence of interaction on the mean error of residuals.

Xie et al.^11^ found that existing UAV trajectory prediction methods are usually affected by external factors and sudden changes in flight mode. Therefore, a framework for UAV trajectory prediction based on Gaussian Process Regression (GPR) is presented. The framework consists of three parts: the first part predicts uncertainty quantification using GPR, the second part detects online track change points, and the third part deals with training data.

Ren et al.^12^ proposes a framework for developing and validating trajectory modeling and prediction methods for various types of UAVs in standard and real-world environments.

The state estimation model establishes motion equations based on the aircraft's position, speed, acceleration and other attributes to achieve the propagation of estimates. The model is relatively simple. However, the state estimation model will lead to large errors due to its inability to accurately capture the maneuvering uncertainty of the aircraft over a long period of time. So it can only work for a short period of time. Although flight intention information has begun to be incorporated into prediction models in order to improve prediction accuracy, the inference of intention is only accurate in the short term.

### Dynamic methods

Schuster et al.^13,14^ adds real-time information to correct aircraft dynamics based on a three-dimensional point mass model of the aircraft. A high performance 4-D trajectory prediction model for civil aircraft is presented. Solves the problem that trajectory pre-diction performance is limited by the precision of predefined settings in existing databases. On this basis, Schuster^15^ combines the existing flight trajectory prediction model with Newton’s laws of physics and aircraft operating procedures to develop a new technology to predict aircraft tracks.

The algorithm proposed by Thipphavong et al.^16^ is also the first to model an air-craft, but it dynamically adjusts the weight of the modeled aircraft based on the observed track data to improve the accuracy of climb flight trajectory prediction.

Lee et al.^17^ derives a stochastic nonlinear mixed model to describe the aircraft behavior during the descent phase. Based on the state-dependent transition hybrid estimation algorithm, a predictive algorithm for aircraft tracking and estimated arrival time (ETA) is presented in the framework of a random nonlinear mixed model. This algorithm can explain continuous state-dependent flight mode transitions.

Zhang et al.^18^ implements an online four-dimensional trajectory prediction (4D-TP) method. Firstly, a 4D-TP model is established based on the dynamic model, air-craft intent, environmental conditions and performance parameters. Then, the ADS-B data is com-pared with the predicted trajectory. When the position or speed deviation exceeds the pre-set threshold, the aircraft intent is updated and the predicted trajectory is re-predicted.

Similarly, Fukuda et al.^19^ builds trajectory prediction models based on aircraft performance, airline operations, navigation databases, and weather forecasts.

Tang et al.^20^ builds a hybrid system model to estimate the 4D trajectory of different types of aircraft based on their flight profiles and dynamic models under different flight conditions.

Baklacioglu et al.^21^ uses a genetic algorithm (GA) to derive a new aeronautical propulsion model (APM) from the flight manual data of a transport aircraft for accurate trajectory prediction.

The point mass model (PMM) is commonly used in dynamic models. Through some simplifying assumptions, the analysis is carried out from the perspective of aircraft force, and Newton's second law is used to derive dynamic equations. Finally, a set of kinematics and dynamic equations are combined to derive Differential equations are composed of equations of motion (EOM). However, dynamic models are mostly implemented under some ideal assumptions and simplified, with little consideration of practical constraints and human behavior. In addition, since information on aircraft performance, aircraft status, environmental conditions, and aircraft intentions are taken into account, the model requires a large number of parameters, some of which are commercially sensitive and not easy to obtain. Once data resources are limited or insufficiently supported, the prediction accuracy of the model will be greatly reduced or even inapplicable.

### Machine learning methods

Tastambekov et al.^22^ are based entirely on historical radar trajectory data and do not use any physical or aeronautical parameters. After these radar data are preprocessed, the aircraft trajectory is predicted by local linear function regression. De Leege et al.^23^ also use a stepwise regression algorithm to predict the arrival time of an aircraft based on historical data. Lin et al.^24^ also used historical flight track data as the source of raw data. However, he predicts flight trajectories based on the Hidden Markov Model (HMM), uses EM algorithm to optimize the parameters of the HMM model, and finally uses the optimized HMM model to predict the 4-D trajectory of the aircraft. Using the last flight plan before takeoff, Pang et al.^25^ proposed a probability aircraft trajectory prediction method based on Bayesian neural network. Wu et al.^26^ studied an aircraft 4-D trajectory prediction model based on BP network, using the airplane broadcast trajectory from Qingdao to Beijing as the data source. It solves the problem that traditional trajectory pre-diction methods cannot meet the requirements of high-precision, multi-dimensional and real-time prediction. Den et al.^27^ proposed a flight trajectory recognition frame-work based on recursive neural networks and validated it by real-time predicting the air-craft's expected arrival time. Wang et al.^28^ used machine learning to predict short-term trajectories of aircraft. They constructed a hybrid model that first preprocessed the data using principal component analysis (PCA) and a clustering algorithm, followed by a multicellular neural network (MCNN) for short-term 4D trajectory prediction.

The above scholars use classic machine learning methods, and deep learning methods have also been widely used in aircraft trajectories. Capturing short-term and long-term relationships, Fernando et al.^29^ propose a tree memory network (TMN) to de-scribe long-term and short-term relationships within sequences and to predict aircraft trajectories in the environment. Ma et al.^30^ In order to solve the problem of path conflict in airplane gliding on the ground, a decay memory window is introduced to improve the hidden layer structure of LSTM by using the correlation between the sequences of gliding positions of the airplane. Finally, the improved LSTM is used to predict the trajectory. Shi et al.^31^ divided the aircraft into three stages: ascent, descent and cruise ac-cording to the dynamic characteristics of the aircraft. Under the constraints of these three stages, a constrained Long Short-Term Memory network model is proposed. This model is used to predict the 4-D trajectory of an aircraft. Aiming at the situation that military air-craft often shield the ADS-B signal, Yang et al.^32^ use passive radar to collect air-craft trajectory data, and use LSTM to predict its future short-term trajectory.

Further, some scholars integrate different deep learning models and propose some hybrid models. Zhang et al.^33^ used two deep learning models, deep neural net-work (DNN) and deep long-term short-term memory (LSTM) neural network, to predict aircraft trajectories, and finally constructed a hybrid model of the two models. Shafienya et al.^34^ proposed a hybrid model based on the Hartsfield–Jackson Atlanta International Airport. The model adopts ADS–B as the input of the model, a hybrid convolutional neural network and gated recurrent unit (CNN-GRU) and a three-dimensional (3D-CNN) model for aircraft prediction. Zhang et al.^35^ constructs a space–time convolution neural network (STG-CNN) model to solve the collision problem during airport ground operations. This model can predict the movement of objects on the extended ground, including airplanes. Since the 4D trajectory of an aircraft includes time dimension and three spatial dimensions, Shafienya et al.^36^ use CNN network to extract spatial features and GRU to extract temporal features to build a hybrid deep learning model. The method uses the preprocessed ADS-B data as a dataset for aircraft trajectory prediction. Similarly, Ma et al.^37^ combines CNN with LSTM to construct a deep learning hybrid model to predict the 4D trajectory of an aircraft. CNN is used to extract spatial features and LSTM is used to extract temporal features.

Since factors such as weather, aircraft characteristics and flight plans can also affect the trajectory of aircraft, some scholars have done study considering the above factors. Pang et al.^38^ combined convolutional layers with LSTMs to predict trajectories based on convective weather conditions in an aircraft's flight plan before takeoff. Later, Pang et al.^39^ proposed a new Conditional Generative Adversarial Network (CGAN) method for the weather-related aircraft trajectory prediction problem.

Georgiou et al.^40^ used Hidden Markov Models for the aircraft TP task, and comprehensively considered factors such as flight plan, local weather, and aircraft characteristics, increasing the dimension of the 4-d trajectory model.

Traditional trajectory predictors require multiple inputs, such as flight plans, aircraft performance models, weather forecasts. Many of these data are affected by environmental uncertainty. Based on Encoder-Decoder Architecture, Tran et al.^41^ build a deep learning model using GRU network without requiring aircraft performance and wind di-rection data. This model is used to predict aircraft trajectory with aircraft intent data. Zeh et al.^42^ Introduces a causal model to deal with the effects of uncertainty on an air-craft's 4D trajectory, including atmospheric conditions, aircraft take-off weight, wind speed, and so on. Also in the field of intelligent transportation, Chen et al.^43^ first conducted study on vehicle trajectories from a microscopic perspective. They matched the vehicle position with the trajectory data output from the previous step through the Kalman filter and the Hungarian algorithm to obtain the vehicle imaging trajectory frame by frame. Later, Chen et al.^44^ studied the problem of short-term traffic flow prediction from a macro perspective. Wavelet filters, moving average models, and Butterworth filters are used to remove anomalies in traffic flow data, and finally artificial neural networks are used to predict short-term traffic flow evolution.

Machine learning models do not require explicit modeling of aircraft performance, procedures and airspace and are built with weak or no assumptions to predict flight trajectories by learning from historical flight trajectories using machine learning and data mining algorithms. This makes it possible to mine complex trajectory patterns and extract important features, and in some cases, machine learning models can show better predictive performance. However, the machine learning model is more like a kind of data engineering, describing only rough features and ignoring the hidden transition patterns of the flight trajectory. Most of the existing data are flight trajectory data obtained through simulation. However, in practice, each aircraft model needs to be simulated, and the parameter changes during the flight of the aircraft also need to be considered, which will cause the data obtained by simulation to be inaccurate. Flawed. The accuracy of the data source will directly affect the machine learning model and lead to flaws in the prediction results.

This study utilizes historical ADS-B data to predict aircraft trajectories in the event of ADS-B failure without explicit modeling of aircraft performance, procedures, and airspace. At the same time, it avoids the problem of frequent dynamic modeling of different types of aircraft under different circumstances.

## Theoretical basis of neural network for predicting aircraft trajectories

### Data preprocessing

**Figure 1:**
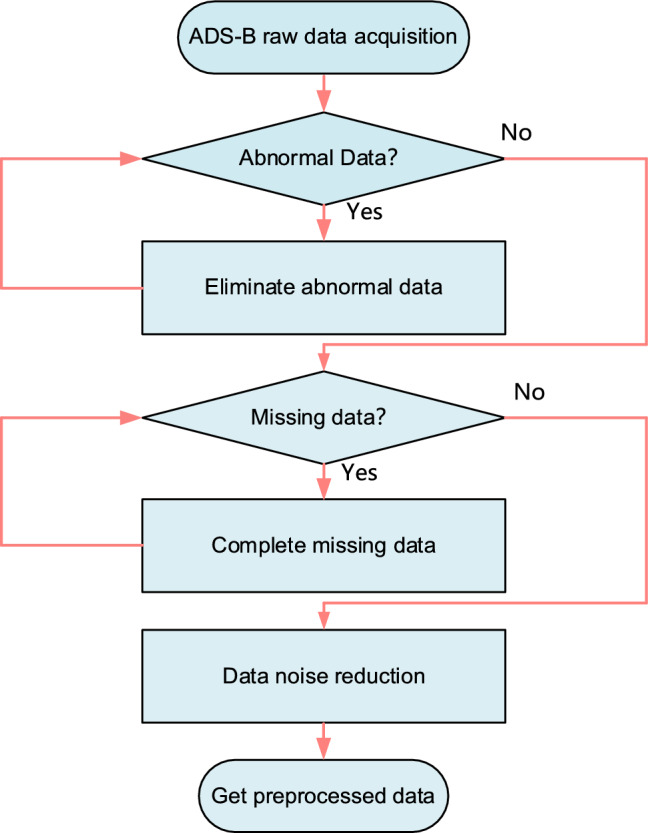
Data preprocessing flowchart.

The detection of abnormal data is a key task that determines the quality of subsequent model training^45^. In the process of data acquisition, data errors may be caused by system failures and sensor errors. The data therefore needs to be preprocessed before it can be used. Data pre-processing consists of the following common forms: handling of blank data, attribute coding, data normalization, and feature selection. Preprocessing is mainly divided into the following three categories: first, eliminate unreasonable data in the dataset, second, fill in the missing data in the dataset, third, reduce the noise of the data. The data preprocessing process adopted in this article is shown in Fig. 1.

#### Abnormal data processing

The longitude, latitude, and altitude data of the aircraft are broadcasted through ADS-B. Abnormal data may be generated during transmission and reception, leading to a decrease in the prediction accuracy of the model. In this paper, the threshold method adopted to process abnormal data, the data within the threshold range is retained, and the data outside the threshold is eliminated.

1 $$ - 180 \le Data\_longitude(j) \le 180 $$

2 $$ - 90 \le Data\_latitude(j) \le 90 $$

3 $$ 0 \le Data\_altitude(j) \le THR $$

where $Data\_longitude(j)$, $Data\_latitude(j)$, $Data\_altitude(j)$ represents the longitude, latitude, and altitude of the aircraft’s ADS-B data at time $j$ respectively, $THR$ represents the maximum flight altitude of the aircraft. If the detected data exceeds the threshold, it means that the observer may have abnormal data in data processing, which needs to be eliminated. Similarly, the altitude should also be greater than 0.

#### Missing data processing

ADS-B data signals may lose data during broadcasting, or data may be lost due to temporary failure of system hardware and software. This can also adversely affect fore-casting, so the time series method is used to complete the lost data.

4 $$ Data_{i} (j) = \frac{1}{2n}\left( {Data_{i} (j - n) + \cdot \cdot \cdot + Data_{i} (j - 1) + Data_{i} (j + 1) + \cdot \cdot \cdot + Data_{i} (j + n)} \right) $$

where $Data_{i} (j)$ represents the longitude ($i = 1$), latitude ($i = 2$), and altitude($i = 3$) of the aircraft’s ADS-B data at time $j$ respectively, $n$ represents selecting $n$ sets of data to complete the data. Considering the time correlation between the lost data and the existing data, the data supplemented by the time series method will also be kept within a reasonable range.

#### Data noise reduction processing

During the observation and transmission of data, it will be interfered with by external noise and produce errors. Therefore, the historical mean method is used to filter and smooth the noise.

5 $$ Data_{i} (j) = \frac{1}{n}\left( {Data_{i} (1) + Data_{i} (2) + \cdot \cdot \cdot + Data_{i} (n)} \right) $$

where $Data_{i} (j)$ represents the longitude ($i = 1$), latitude ($i = 2$), and altitude($i = 3$) of the aircraft’s ADS-B data at time $j$ respectively, $n$ represents selecting $n$ sets of data to complete the data.

### LSTM

The general neural network is a fully connected network from the input layer to the hidden layer and then to the input layer. There are no connections between nodes in each layer. In the case of a CNN network, the data it processes is generally stand-alone, identically distributed (IID). These data are entered individually in a CNN network, and there is no relationship between the previous input and the latter. Generally speaking, the neural network represented by CNN can better handle classification, regression and feature ex-pression problems. For example, the classic recognition of cats and dogs, handwritten numbers and so on.

However, in practical applications, some tasks need to be able to better process sequence information, that is, the input before and the input after are related. Typical tasks include language translation, automatic text generation, time series data prediction, and so on. The data in these tasks is data collected at different points in time, reflecting the changing state or degree of something, phenomenon, etc. over time. Therefore, these data do not meet the requirements of IID.

**Figure 2:**
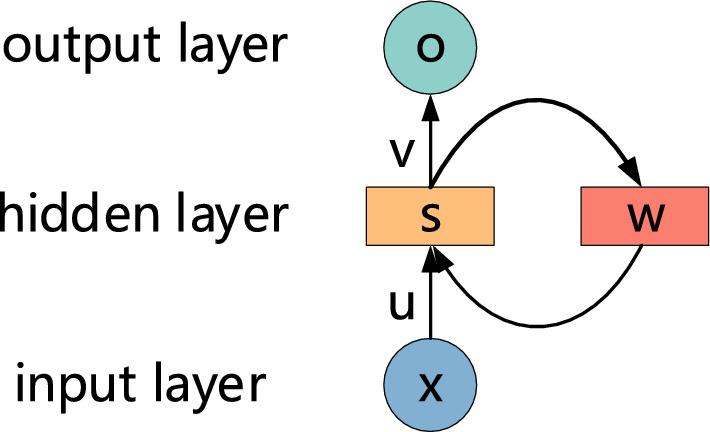
The unit structure of the RNN.

For these tasks, algorithms such as CNN perform poorly, so RNN came into being. In many neural networks, RNNs are better suited for processing time series data. Because RNN contains not only the traditional process of input to hidden layer and hidden layer to output, but also the process of transfer from hidden layer to hidden layer, which is shown in Fig. 2. It is clear that the output of the hidden layer depends not only on the input but also on the output of the hidden layer from the previous moment. Therefore, RNNs memorize the previous information and apply it to the calculation of the current output. Compared with CNN, nodes between hidden layers in RNN are connected.

Considering RNN also has some problems, such as the disappearance of gradients. Therefore, LSTM is proposed, and this network structure is also used in this article.

In order to solve the aforementioned gradient vanishing problem, LSTM is proposed. RNN is a chain structure. Each node in RNN uses the same parameters, and the output of the node is determined only by the weight, bias, and activation function. Compared to RNN, LSTM are composed of a series of LSTM Units, which also use a chain structure. But LSTM solves the problem of gradient vanishing of RNN because LSTM introduces a gate mechanism. This mechanism is used to control the circulation and loss of features. Com-pared to the hidden state of the original RNN, LSTM adds a cell state. This is the biggest difference from RNN.

**Figure 3:**
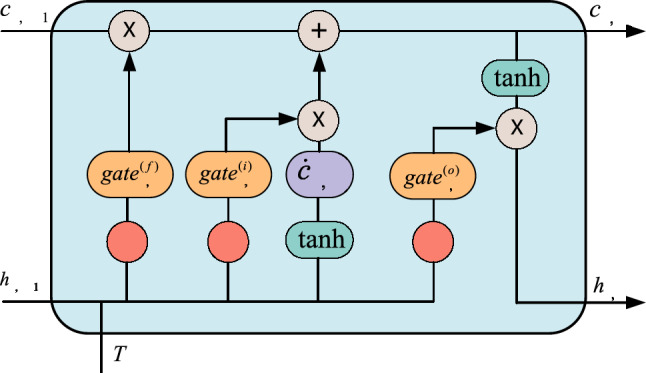
The network structure of LSTM.

The basic unit of LSTM has 3 gate structures, namely the forget gate, the update gate and the output gate. The function of the forgetting gate is to decide what information should be discarded or retained. The information from the previous hidden state and the current input information are passed to the sigmoid function at the same time, and the output value is between 0 and 1. The closer to 0 means the more it should be discarded, and the closer to 1 means the more it should be kept. Update gates are used to update the LSTM cell state to determine what information from the current input is important and needs to be added. The output gate is used to determine the value of the next hidden state, which contains the previously entered information. Figure 3 shows the network structure of LSTM.

From the Fig. 3, it can be seen that the calculation results $h_{\gamma ,\beta }$ of the LSTM neural unit not only propagate between the upper and lower layers, but also between the units in this layer. The unit state $c_{\gamma ,\beta }$ also propagates between the units in this layer, and the memory features are controlled through forgetting gates, which enables LSTM to remember some features of historical time series data when processing data. Equations (6)–(11) is the calculation process for LSTM.

6 $$ gate_{\gamma ,\beta }^{(f)} = \sigma (W^{(f)} (h_{\gamma ,\beta - 1} ,T) + b_{\gamma ,\beta }^{(f)} )\beta $$

7 $$ gate_{\gamma ,\beta }^{(i)} = \sigma (W^{(i)} (h_{\gamma ,\beta - 1} ,T) + b_{\gamma ,\beta }^{(i)} )\beta $$

8 $$ \dot{c}_{\gamma ,\beta } = \tanh (W^{(c)} (h_{\gamma ,\beta - 1} ,T) + b_{\gamma ,\beta }^{(c)} ) $$

9 $$ c_{\gamma ,\beta } = gate_{\gamma ,\beta }^{(f)} * c_{\gamma ,\beta - 1} + gate_{\gamma ,\beta }^{(i)} * \dot{c}_{\gamma ,\beta - 1} $$

10 $$ gate_{\gamma ,\beta }^{(o)} = \sigma (W^{(o)} (h_{\gamma ,\beta - 1} ,T) + b_{\gamma ,\beta }^{(o)} ) $$

11 $$ h_{\gamma ,\beta } = gate_{\gamma ,\beta }^{(o)} * \tanh (c_{\gamma ,\beta } ) $$

among them, $gate_{\gamma ,\beta }^{(f)}$, $gate_{\gamma ,\beta }^{(i)}$ and $gate_{\gamma ,\beta }^{(o)}$ are the forgetting gate, input gate and output gate of the $\beta$ neural unit in the $\gamma$ layer of LSTM respectively, $\dot{c}_{\gamma ,\beta }$ is the unit state of the current neural unit, $c_{\gamma ,\beta }$ is the final unit state of the current neural unit, the output unit state, $h_{\gamma ,\beta }$ is the calculation result of the current neural unit, $\sigma$ is the activation function; $W$ is the weight matrix; $b$ is the bias matrix; $(h_{\gamma ,\beta } ,T)$ is the input.

### Bi-LSTM

**Figure 4:**
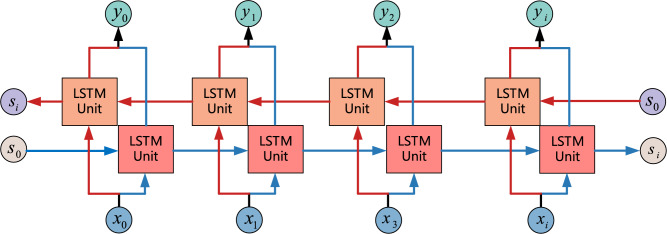
The network structure of Bi-LSTM.

LSTM can capture long-term dependencies in sequence data for time series estimation, but it only uses information from previous states^46^. However, sometimes predictions may need to be determined jointly by several inputs from the front and several inputs from the back, which will be more accurate. Therefore, a bidirectional recurrent neural network is proposed, and the network structure is shown in Fig. 4. It can be seen that the Forward and Backward layers are jointly connected to the output layer.

The Bi-LSTM neural network structure model is divided into two independent LSTMs. The input sequence is input into two LSTM neural networks in positive and reverse order for feature extraction, and the two output vectors are concatenated to form the final feature expression. The model design concept of Bi-LSTM is to enable the feature data obtained at time t to have both past and future information.

## Aircraft trajectory prediction framework

### Aircraft ADS-B data sources

**Figure 5:**
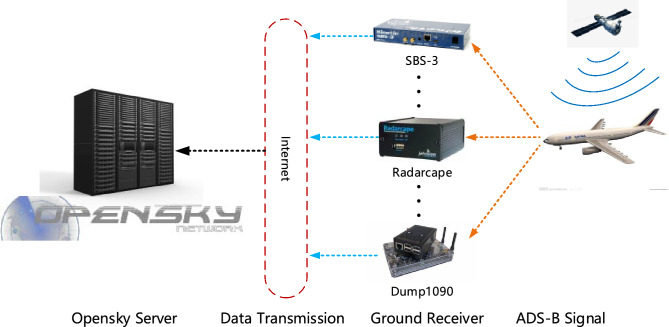
Schematic diagram of Opensky’s network transmission/data collection.

The dataset of this article comes from the aircraft ADS-B data publicly provided by OpenSky. The Opensky system has many volunteers around the world. These volunteers receive ADS-B data from the aircraft and transmit the information to Opensky’s servers. The network transmission/data collection of Opensky is shown in Fig. 5.

The data is provided in CSV format. There are two files, one containing measurement data information and the other containing information about sensor data. Measurement data information is used as the data source here, as shown in Table 1. The original data file contains information such as ID, server time, aircraft identifier, latitude and longitude, altitude, and number of receivers. The longitude and latitude in the original data are in the WGS84 coordinate system. The expression geoaltitude comes directly from the original ADS-B data of the aircraft. It is one of the data categories reported by the aircraft to the outside world and represents the geographical altitude reported by the aircraft.

**Table 1:** Sample of ADS-B raw data message from Opensky.

ID	timeAtServer	Aircraft	Latitude	Longitude	geoAltitude	Num-Measurement
1	0	304	52.37887573	0.659866333	6286.5	4
2	0	2671	42.28720093	1.798137318	8915.4	4
3	0	1940	47.05220032	5.903640747	10,820.4	2
4	0.001	843	51.62434387	5.030699034	9959.34	14
5	0.002	1843	50.82042629	3.699264526	2080.26	3
…	…	…	…	…	…	…
12	0.003	2007	NaN	NaN	NaN	9
…	…	…	…	…	…	…
1005	0.477	2671	42.28784	1.797576	8915.4	3
…	…	…	…	…	…	…
1011	0.479	1843	50.81992	3.700017	2072.64	2
…	…	…	…	…	…	…
10,179	5.496000051	2671	42.29610637	1.79134635	8915.4	3
…	…	…	…	…	…	…

In the ADS-B raw data message, it can be seen that the yellow part in the table represents part of the ADS-B data of aircraft number 2671, and the blue part represents part of the ADS-B data of aircraft number 1843. At the same time, it can also be directly observed that some data are missing. The causes of the problem include the operation level of volunteers, the reliability of data transmission, the strength of the signal, the quality of network transmission and other factors.

### Prediction framework

**Figure 6:**
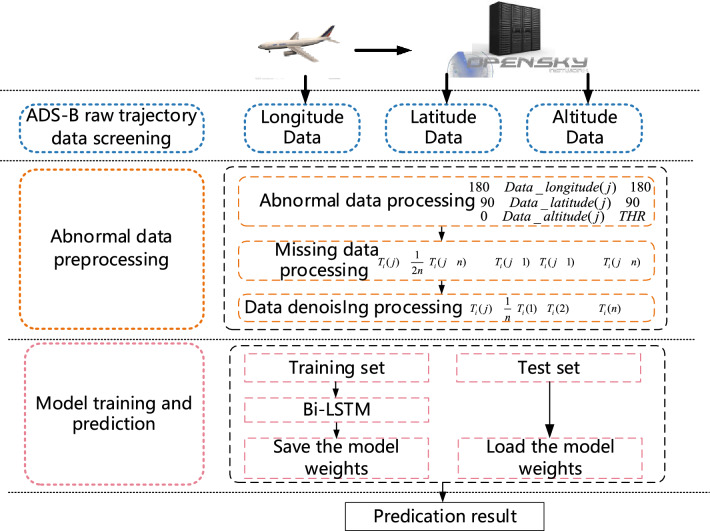
Aircraft trajectory prediction framework.

The prediction framework as shown in Fig. 6.

The prediction framework is divided into three parts. The first part is the screening of ADS-B raw data, which is mainly used to select flight tracks with relatively complete tracks; The second part is data preprocessing, mainly used to further process the screened trajectories and reduce the interference of abnormal data; The third part is the training of the model and the prediction of data, mainly by constructing Bi-LSTM to predict trajectories.

#### ADS-B raw trajectory data screening

Since the ADS-B data comes from the GPS system, it is not the aircraft flight control system that independently calculates its position, altitude, speed and other important navigation information. The GPS system is vulnerable to intentional or unintentional illegal interference. This will cause the accuracy and integrity of ADS-B data derived from GPS system data to be reduced, or even fail to meet the standards required for operation^47^.

Australia is the first country among the members of the International Civil Aviation Organization (ICAO) to announce the full adoption of ADS-B for airspace surveillance. The ADS-B information released one after another by the country’s air traffic control department points out that there is discontinuity in the ADS-B track. In March 2019, Federal Aviation Administration (FAA) released an ADS-B performance user guidance report, requiring users to report ADS-B data quality and use the Navigation Position Accuracy Classification Value (NACP) to indicate ADS-B availability. It can be seen that the FAA has also begun to worry about ADS-B data quality issues. At present, discontinuities and jumps are common in ADS-B tracks, which has led to doubts about its air traffic control service capabilities^48^.

**Figure 7:**
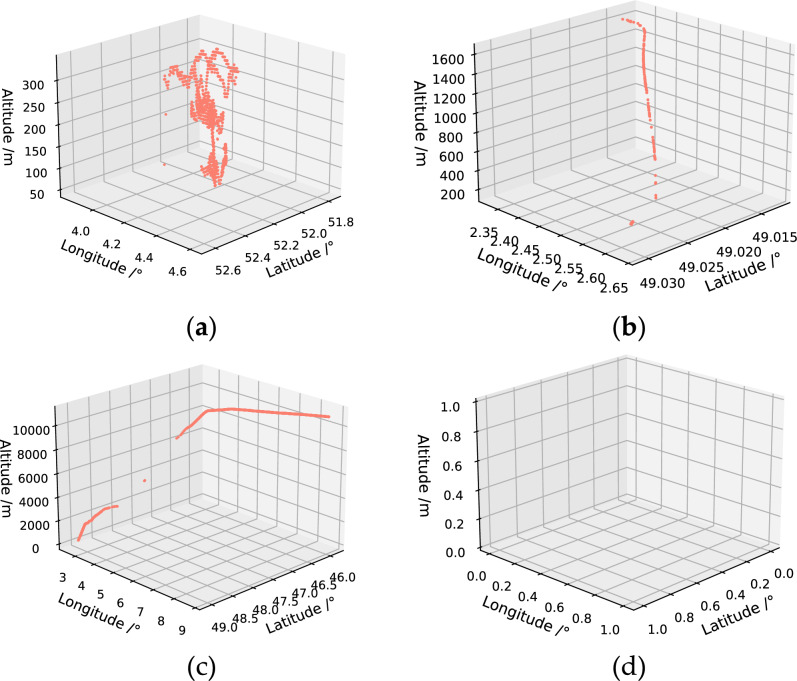
Sample of ADS-B raw data with significant deviation: (**a**) Damaged trajectory; (**b**) Intermittent trajectories; (**c**) Signal interrupted trajectory; (**d**) Signal free trajectory.

As shown in Table 1, the ADS-B signal in the raw data file can be interfered, interrupted, or corrupted. The trajectories resemble Fig. 7a–d.

**Figure 8:**
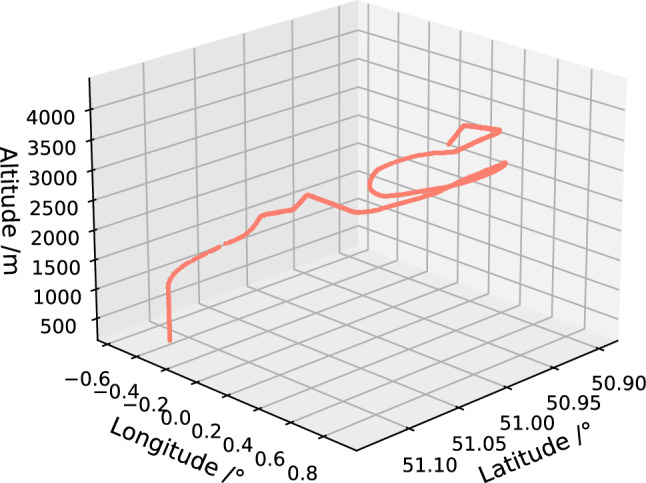
Relatively complete aircraft trajectories after screening.

There is a significant deviation between ADS-B data and the actual position of the aircraft, which is manifested as large-scale position deviation, position rebound, Z-shaped oscillation, altitude jump, etc. If the problematic data shown in Fig. 7 is used, it will result in poor training effectiveness. Therefore, it is necessary to first conduct data screening to select relatively complete aircraft trajectory data for subsequent model training. This article first visualizes the ADS-B raw data, and then performs preliminary manual screening to eliminate seriously damaged data, such as blank data, large-scale damaged data, etc. The relatively complete trajectory screened out is shown in Fig. 8.

#### Abnormal data preprocessing

**Figure 9:**
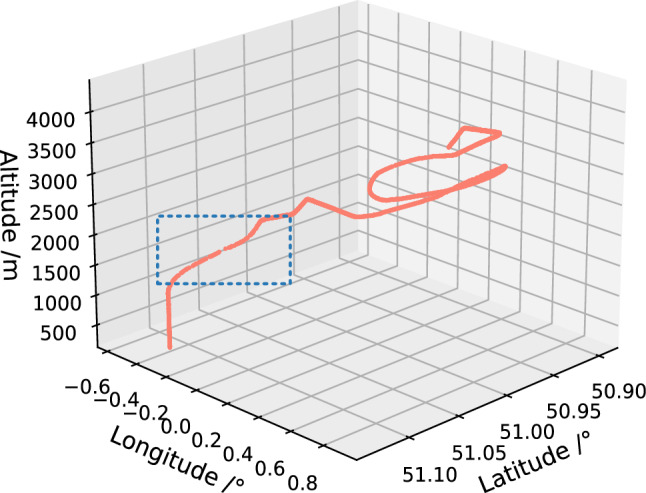
Abnormal data still exist in relatively complete aircraft trajectories after screening.

After preliminary screening of relatively complete aircraft trajectory, it can be observed in Fig. 9 that the trajectory still have problems in some areas. Therefore, the method described in section "Data preprocessing" was used for further processing of the data.

The data preprocessing process adopted in this article is as follows: first, the thresh-old method is adopted to eliminate possible error data, such as negative numbers, data that exceeds the threshold too much, etc.; second, the missing data is completed using the time series method until the missing data no longer exists in the data; finally, the data is noise-reduced using the historical mean method to eliminate possible interference noise in the data. The details of trajectory screened and data preprocessing are shown in Table 2.

**Table 2:**
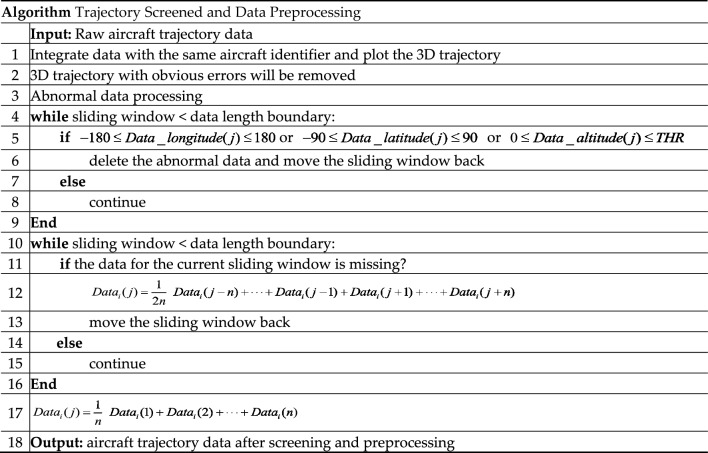
Details of trajectory screened and data preprocessing.

#### The construction of Bi-LSTM

This article selects a bidirectional LSTM neural network based on the characteristics of experimental samples and the requirements for predictive timeliness. Avoid problems such as underfitting, gradient vanishing, and gradient explosion in network training, and achieve good training results.

The number of hidden layer nodes is directly related to the complexity and output accuracy of the solution. When the number of nodes is too large, the network generalization ability will be lost, and even the phenomenon of “overfitting” will appear; When the number of nodes is too small, it will cause a decrease in training ability and poor performance^30^. Therefore, selecting networks with different numbers of nodes for prediction to find the optimal network structure.

**Figure 10:**
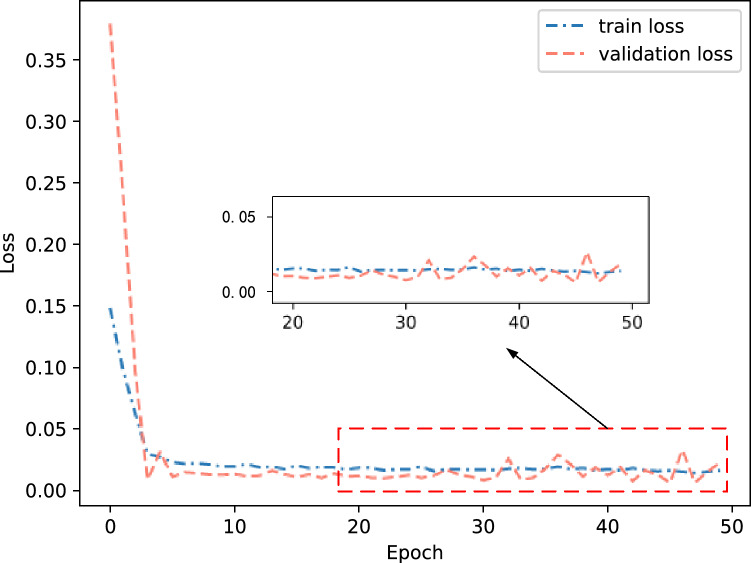
Training error under different training times.

**Figure 11:**
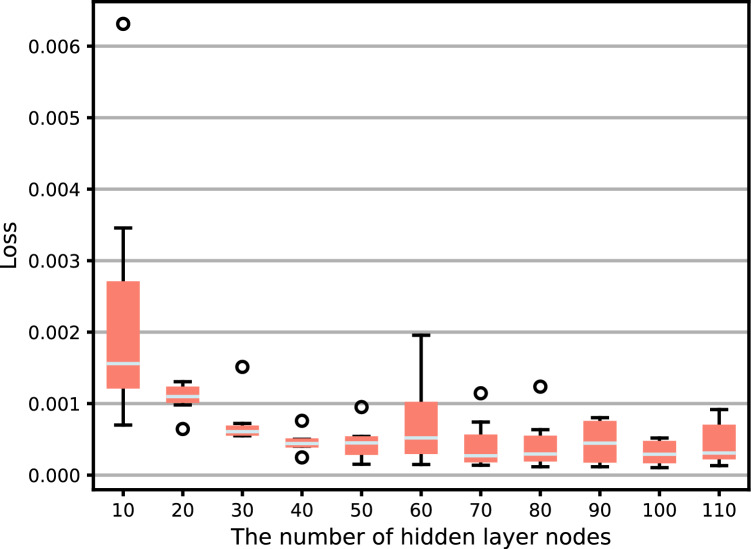
Training error under different numbers of hidden layer neurons.

This article measures parameters such as the number of model training sessions and the number of hidden layer neurons using mean square error (MSE) to determine the parameter combination with better prediction performance. Firstly, the preset number of training iterations is 50, and the number of hidden layer neurons ranges from 10 to 110. The results are shown in Figs. 10 and 11.

**Figure 12:**
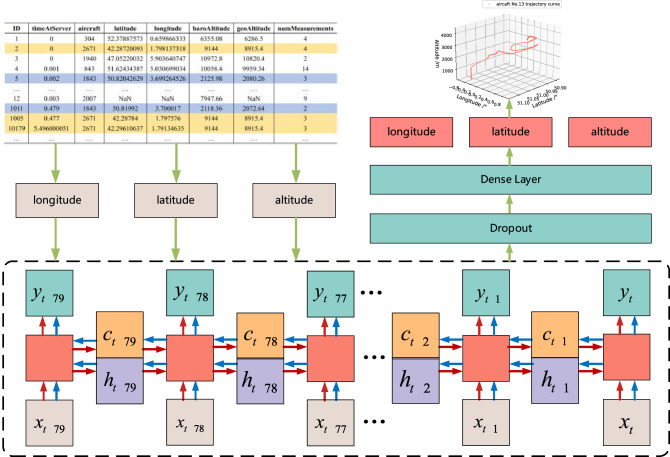
Composition of aircraft trajectory prediction framework.

From Fig. 10, it can be seen that when epoch is 30 times, the loss value is the smallest. As the number of epochs continues to increase, the loss value begins to oscillate and rise. Therefore, the epoch selection for the network is 30. From Fig. 11, it can be seen that the number of nodes in the hidden layer increases from 10, while the loss begins to decrease. But when the number of nodes increases to 80, the loss is the most stable and minimum. Therefore, the number of nodes selected for the dual hidden layer network is 80. The activation function is ReLu, and the learning rate is 0.001. Therefore, the specific structure of the framework is shown in Fig. 12.

## Experiment

### Evaluation indicators

In this article, three evaluation indexes: mean squared error (MSE), mean absolute error (MAE), and mean absolute percentage error (MAPE) were selected to evaluate the predicted trajectories in the experiment. The three indicators can evaluate the performance of the model from three different aspects, which is more comprehensive.

MSE is used to calculate the mean of the sum of squares of the corresponding point errors between the predicted data and the original data. The smaller the value of MSE, the better the accuracy of the prediction model. The calculation formula for MSE is as follows:

12 $$ MSE = \frac{1}{n}\sum\nolimits_{j = 1}^{n} {[T(j) - \overline{R} (j)]^{2} } $$

where $T(j)$ is real value at time $j$, $\overline{R} (j)$ is predicted value at time $j$.

MAE is the mean absolute error, which represents the average of the absolute error between the predicted value and the observed value. The smaller the value of MAE, the better the accuracy of the prediction model in describing experimental data. The calculation formula for MAE is as follows:

13 $$ MAE = \sum\nolimits_{j = 1}^{n} {\left| {T(j) - \overline{R} (j)} \right|n^{ - 1} } $$

where $T(j)$ is real value at time $j$, $\overline{R} (j)$ is predicted value at time $j$.

MAPE is average absolute percentage error. Although MSE can characterize the deviation between the predicted value and the true value, if there are outliers, the MSE will perform poorly. Compared with MSE, MAPE is equivalent to normalizing the error of each point, reducing the influence of absolute error caused by individual outliers. Therefore, MAPE is a more robust indicator than MSE. The calculation formula for MAPE is as follows:

14 $$ MAPE = \frac{100\% }{n}\sum\limits_{j = 1}^{n} {\left| {\frac{{\widehat{y}_{j} - y_{j} }}{{y_{j} }}} \right|} $$

where $y_{i}$ is real value at time $j$, $\widehat{y}_{i}$ is predicted value at time $j$

### Experimental deployment

In the event of on-board equipment failure and local area signal interference, the ADS-B signal will disappear or be interrupted in ADS-B terminal, which will bring danger to aviation safety.

This article simulates this situation using Opensky’s ADS-B data. Use the first 70% of the aircraft trajectory data as historical data that can be obtained during ADS-B normal status, and remove the remaining 30% of the data from the overall data to simulate the scenario after ADS-B failure.

Afterwards, the first 70% of historical ADS-B data will be selected and preprocessed to train the Bi-LSTM in the trajectory prediction framework. Finally, compare the remaining 30% predicted trajectory with the actual trajectory.

### Comparison of experimental results

To improve the prediction accuracy, this article independently predicts the three-dimensional coordinates, and compares the errors with other prediction methods.

**Figure 13:**
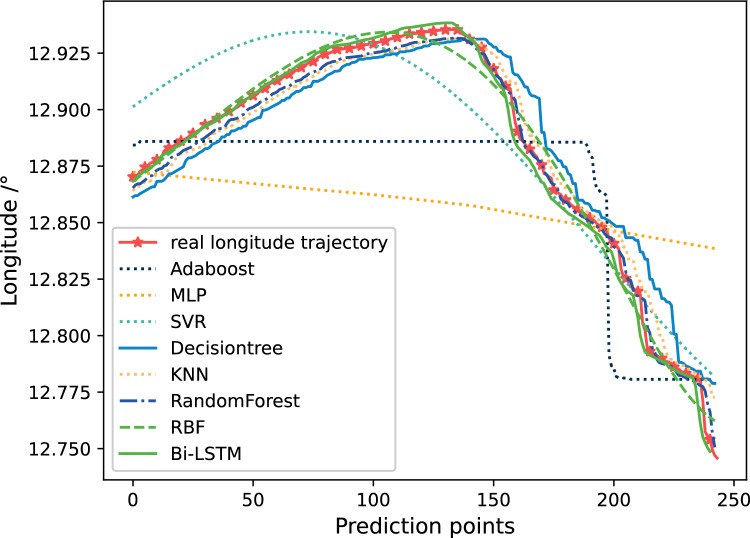
The comparison of prediction results of 7 methods on longitude trajectories.

Figure 13 shows the predicted trajectory of longitude. This figure shows the comparison between the prediction results based on Bi-LSTM and those of other algorithms. From Fig. 13, it can be seen that compared to ordinary neural networks and traditional prediction models, Bi-LSTM’s prediction results are closer to the original data, and there are indeed certain advantages in the accuracy of prediction, with smaller fluctuations than the comparison algorithm. The predicted results of Adaboost, MLP, and SVR differ greatly from the actual situation, and can only predict the approximate direction.

**Figure 14:**
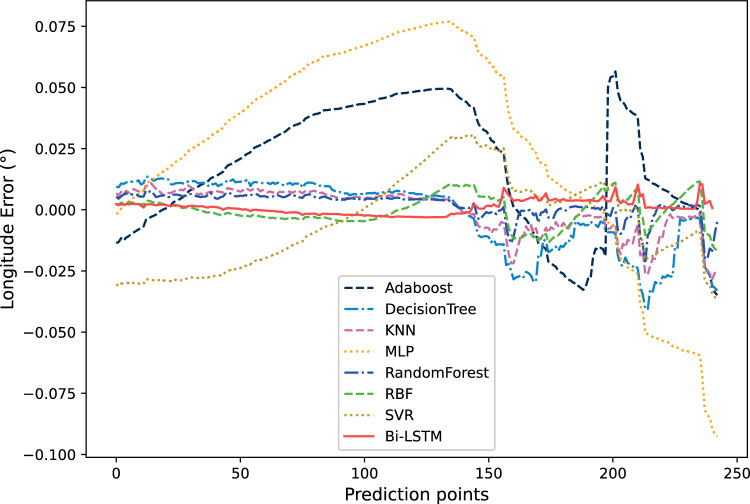
The comparison of relative errors in longitude prediction results.

Figure 14 shows the comparison of relative errors in longitude prediction results. It can be seen that the error of Bi-LSTM represented by the solid red line is closer to 0. This can also prove that Bi-LSTM not only can obtain more accurate prediction results com-pared to ordinary neural networks, but also has more stable prediction performance. And from Fig. 14, it can be seen that the relative prediction errors of Adaboost, MLP, and SVR are also relatively large, which is consistent with the results shown in Fig. 13.

**Figure 15:**
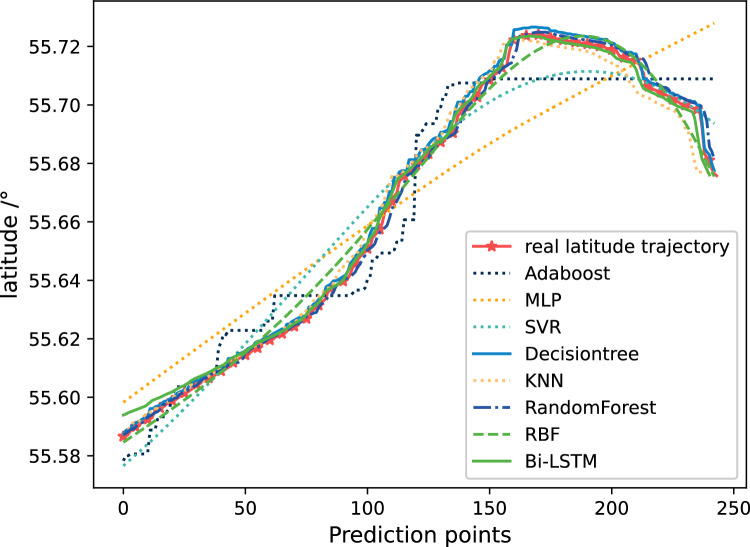
The comparison of prediction results of 7 methods on latitude trajectories.

Figure 15 shows the trajectory prediction results of latitude. This figure showing the comparison between the prediction results based on Bi-LSTM and those of other algorithms. From Fig. 15, it can be seen that the predicted results of Adaboost, MLP, and SVR still differ significantly from the actual trajectory. The prediction results of Bi-LSTM have a certain deviation at the prediction point around 0–40, but as the prediction continues, the deviation is gradually narrowing. After the prediction point 40, the prediction results of Bi-LSTM are relatively close to the actual trajectory curve. The prediction results of Decision Tree and Random Forest for latitude are different from those for longitude, and the prediction results for latitude are also very close to reality.

**Figure 16:**
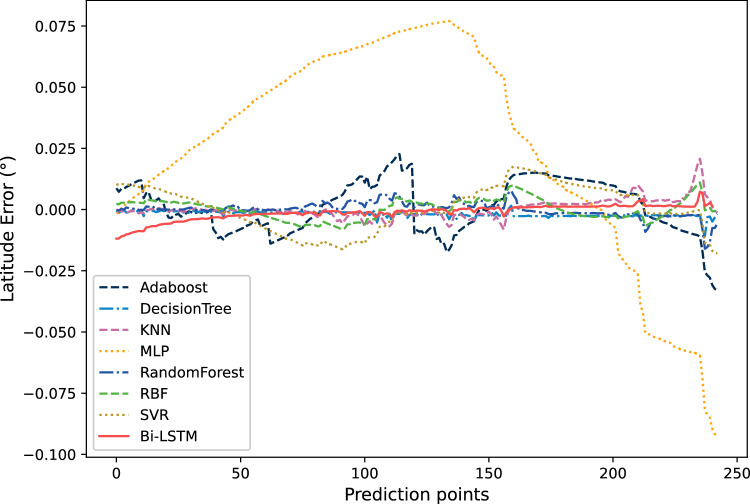
The comparison of relative errors in latitude prediction results.

Figure 16 shows the comparison of relative errors in latitude prediction results. It can be seen that the error of Bi-LSTM represented by the red solid line is closer to 0 after the prediction point 40, with only a few positions experiencing fluctuations in the middle. At the same time, the relative prediction error of Decision Tree and Random Forest is also very small.

**Figure 17:**
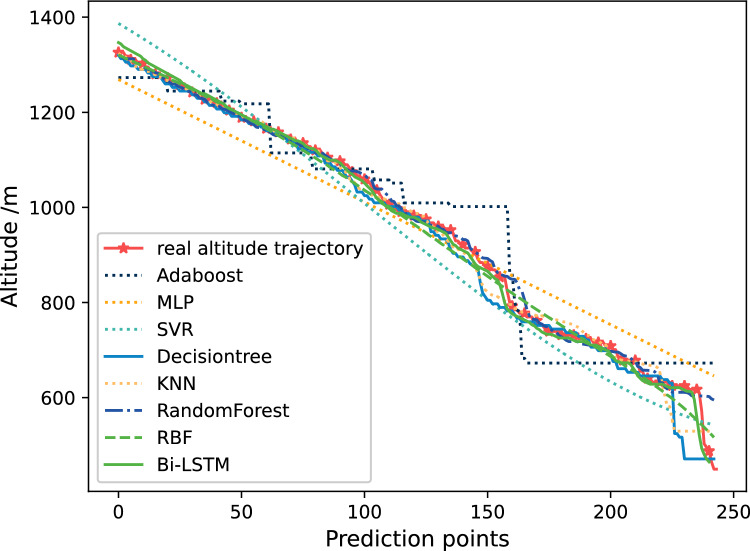
The comparison of prediction results of 7 methods on altitude trajectories.

Figure 17 shows the altitude trajectory prediction results. This figure shows the comparison between the prediction results based on Bi-LSTM and those of other algorithms. From Fig. 17, it can be seen that except for Adaboost, MLP, and SVR, most algorithms can perform predictions well. Among them, DecisionTree has obvious errors at about 150 and 230 prediction points. However, compared with ordinary neural networks and traditional prediction models, Bi-LSTM's prediction results are very close to the original data, and its prediction accuracy has certain advantages with small fluctuations.

**Figure 18:**
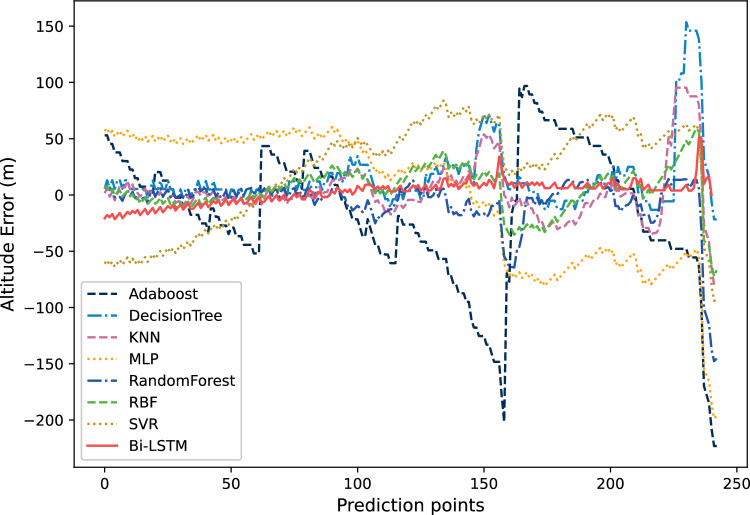
The comparison of relative errors in altitude prediction results.

Figure 18 shows the comparison of relative errors in altitude prediction results. The relative error of Adaboost at prediction points 60, 160, and 230 is very large, which also corresponds to the results in Fig. 17. The relative error between MLP and SVR is also relatively large, especially after the prediction point 160. It can be seen that the error of the red solid line Bi-LSTM is closer to 0, except for the first 30 prediction points. This can also prove that Bi-LSTM not only can obtain more accurate prediction results compared to ordinary neural networks, but also has more stable prediction performance.

In addition to qualitative analysis using trajectory and error maps, this article also uses three evaluation indicators: MSE, MAE, and MAPE to quantitatively evaluate the prediction results of the algorithm. The results are shown in Tables 3, 4, and 5, respectively.

**Table 3:** The comparison of prediction errors in latitude.

Evaluation indicators	Latitude							
	Adaboost	Decision Tree	KNN	MLP	Random Forest	RBF	SVR	Bi-LSTM
MSE	0.000118	**0.000004**	0.000015	0.000308	0.000009	0.000017	0.000085	**0.000011**
MAE	0.009193	**0.001901**	0.002638	0.015411	0.002015	0.003440	0.007731	**0.002264**
MAPE	0.000165	**0.000034**	0.000047	0.000277	0.000036	0.000062	0.000139	**0.000041**

Significant values are in bold and bold underline.

**Table 4:** The comparison of prediction errors in longitude.

Evaluation Indicators	Longitude							
	Adaboost	Decision Tree	KNN	MLP	Random Forest	RBF	SVR	Bi-LSTM
MSE	0.000945	0.000215	0.000084	0.002521	0.000031	0.000037	0.000376	**0.000009**
MAE	0.026065	0.012394	0.007666	0.043486	0.004397	0.004957	0.016587	**0.002379**
MAPE	0.002021	0.000963	0.000596	0.003373	0.000341	0.000385	0.001287	**0.000185**

Significant values are in bold underline.

**Table 5:** The comparison of prediction errors in altitude.

Evaluation Indicators	Altitude							
	Adaboost	Decision Tree	KNN	MLP	Random Forest	RBF	SVR	Bi-LSTM
MSE	3909.117	1067.418	710.6956	3384.724	587.2740	429.7735	2511.523	**111.0655**
MAE	47.23589	17.93902	16.33182	51.37228	11.91572	15.92314	46.09662	**8.447393**
MAPE	0.059425	0.023337	0.022481	0.062296	0.017273	0.020344	0.053616	**0.009493**

Significant values are in bold underline.

From the table, it can be seen that in the predicted results, except for the latitude pre-diction results, Decision Tree is slightly smaller than Bi-LSTM, and the indicators of Bi-LSTM are far superior to other prediction algorithms in all other dimensions. Therefore, it indicates that the method proposed in this article can effectively solve the problem of flight trajectory prediction accuracy for aircraft.

**Figure 19:**
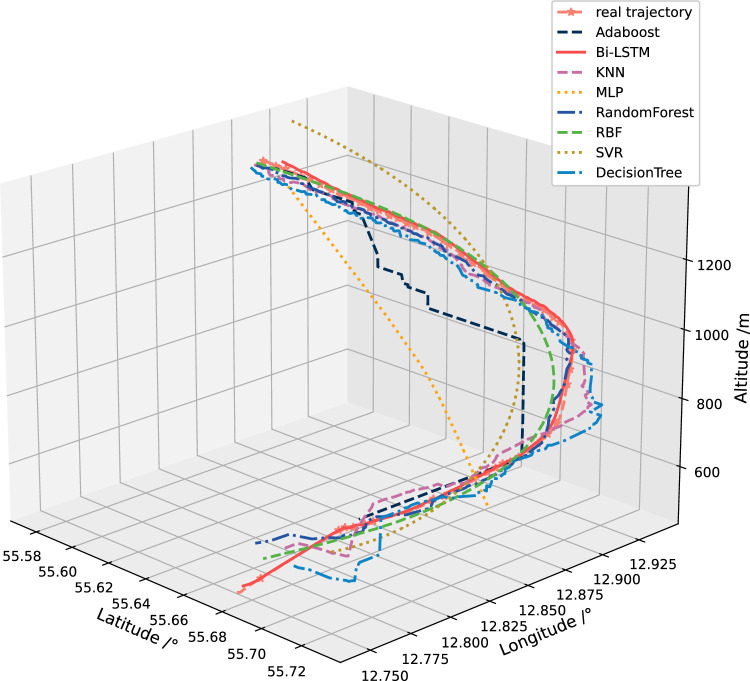
The comparison results between the actual 3D trajectory and the predicted 3D trajectory.

Figure 19 shows the comparison results between the actual 3D trajectory and the predicted 3D trajectory. It can also be seen in the figure that the predicted results based on Bi-LSTM are closest to the actual trajectory. Although in Table 3, the error of Decision Tree is slightly smaller than Bi-LSTM. However, combining Fig. 19, it can be seen that after comprehensive comparison of 3D trajectories, it can be found that the final prediction effect of Bi-LSTM is the best.

## Conclusions

In the event of on-board equipment failure and local area signal interference, the ADS-B's signal will disappear or be interrupted. This sudden situation can pose a danger to aviation safety. To solve this problem, this article proposes Bi-LSTM network prediction method combining historical ADS-B data to short-term predict the trajectory of aircraft, which can improve aviation safety in busy airspace. The conclusions are as follows:

1. This article proposes using historical ADS-B data to predict the trajectory of aircraft in the event of ADS-B failure. It solves the problem of frequent dynamic modeling of different types of aircraft, and improves the extensibility of the method. At the same time, this article proposes a data cleaning method to process ADS-B raw data. It solves the problem that the original ADS-B data has some wrong data, which is not conducive to model training. It can provide a high-quality data source for the trajectory prediction model in this article. Finally, this article proposes a Bi-LSTM aircraft trajectory prediction framework, which can predict aircraft trajectories in the event of ADS-B failure and improve aviation safety in sudden situations. Experiments show that compared with ordinary neural networks and traditional prediction models, the prediction results of this method are closer to the original data, the pre-diction accuracy does have some advantages, and the fluctuation is less than that of the comparison algorithm.
2. This includes establishing an ADS-B operation monitoring agency, responsible for collecting the capability and maintenance status of aircraft ADS-B airborne equipment, ADS-B technical performance monitoring, ADS-B standardized operation status, and regularly or in real time reporting relevant situations and existing problems to all relevant parties. At the same time, continuous monitoring and tracking of problem solving and subsequent operation status are carried out to timely improve and standardize ADS-B operation work, enhance ADS-B operation safety and efficiency. Intensify the promotion and training of ADS-B operations to promote the standardized operation of airlines. Strengthen the standardization of cabin operation for crew members. Continuously track and strengthen study on ADS-B technical standards and operational specifications, promptly identify technical issues and defects in the functionality and performance of ADS-B ground and airborne equipment, and make timely corrections; Improve the operating procedures according to the operating environment and make timely adjustments.

## Data Availability

The datasets generated during and/or analysed during the current study are available from the corresponding author on reasonable request.
